# Sobolev-to-Lipschitz property on ${\mathsf {QCD}}$-spaces and applications

**DOI:** 10.1007/s00208-021-02331-2

**Published:** 2021-12-23

**Authors:** Lorenzo Dello Schiavo, Kohei Suzuki

**Affiliations:** 1 IST Austria, Am Campus 1, 3400 Klosterneuburg, Austria; 2 Fakultät für Mathematik, Universität Bielefeld, 33501 Bielefeld, Germany

**Keywords:** Quasi curvature-dimension condition, Sub-Riemannian geometry, Sobolev-to-Lipschitz property, Varadhan short-time asymptotics, Primary 46E36, Secondary 53C17

## Abstract

We prove the Sobolev-to-Lipschitz property for metric measure spaces satisfying the quasi curvature-dimension condition recently introduced in Milman (Commun Pure Appl Math, to appear). We provide several applications to properties of the corresponding heat semigroup. In particular, under the additional assumption of infinitesimal Hilbertianity, we show the Varadhan short-time asymptotics for the heat semigroup with respect to the distance, and prove the irreducibility of the heat semigroup. These results apply in particular to large classes of (ideal) sub-Riemannian manifolds.

## Introduction

In [20], Milman introduced the notion of *quasi curvature-dimension condition* ${\mathsf {QCD}}$ for a metric measure space $(X,{\mathsf {d}},{\mathsf {m}})$, simultaneously generalizing Lott–Villani–Sturm’s *curvature-dimension condition* ${\mathsf {CD}}(K,N)$ with finite *N* [19, 28, 29], and the *measure contraction property* ${\mathsf {MCP}}$ [22, 29]. As discussed in [20], the class of ${\mathsf {QCD}}$ spaces notably includes large families of (ideal) sub-Riemann manifolds, thus aiming to provide a unified perspective of (non-smooth) Riemannian, Finsler, and sub-Riemannian geometry.

In this note, we collect some metric-measure properties of a metric measure space $(X,{\mathsf {d}},{\mathsf {m}})$ satisfying the ${\mathsf {QCD}}$ condition. As a main result, Theorem 3.6, we show the Sobolev-to-Lipschitz property, see  below.

In light of recent developments in metric analysis, the property  has turned out to be significant in relating differentiable and metric measure structures. For instance,

- under , the Bakry–Émery (synthetic Ricci) curvature (lower) bound ${\mathsf {BE}}$ is equivalent to the Riemannian curvature-dimension condition ${\mathsf {RCD}}$, Ambrosio–Gigli–Savaré [3]. The statement is sharp, in the sense that ${\mathsf {BE}}$ without  does not imply ${\mathsf {RCD}}$, Honda [15];
- together with , the ${\mathsf {BE}}$ condition implies the $L^\infty$-to-Lipschitz regularization of the heat semigroup, Ambrosio–Gigli–Savaré [2] (in the sub-Riemannian setting see Stefani [26]);
- together with a Rademacher-type property for $(X,{\mathsf {d}},{\mathsf {m}})$, see $(\mathsf {Rad})$ below,  implies the coincidence of the intrinsic distance and the given distance ${\mathsf {d}}$, and also implies the integral Varadhan short-time asymptotic for the heat semigroup in a variety of settings (see [9, Thm. 4.25]), and furthermore, for the space of configurations (i.e., locally finite integer-valued point measures) over *X*, see [8, Thm. 6.10].

Apart from , ${\mathsf {QCD}}$ spaces satisfy the local volume doubling, the Rademacher-type property $(\mathsf {Rad})$, and the local versions $(\mathsf {Rad})_{\mathrm {loc}}$ and $(\mathsf {SL})_{\mathrm {loc}}$ of $(\mathsf {Rad})$ and  after [9], see Sect. 4. When $(X,{\mathsf {d}},{\mathsf {m}})$ is additionally infinitesimally Hilbertian, as an application of the Sobolev-to-Lipschitz property, we obtain:

- the coincidence of the distance ${\mathsf {d}}$ with the intrinsic distance ${\mathsf {d}}_{\mathsf {Ch}_{}}$ of the Cheeger energy $\mathsf {Ch}_{}$ of $(X,{\mathsf {d}},{\mathsf {m}})$, Theorem 4.8;
- the integral Varadhan short-time asymptotic for the heat semigroup with respect to the Hausdorff distance induced by ${\mathsf {d}}$, Theorem 4.5;
- in the compact case, and assuming as well the measure contraction property ${\mathsf {MCP}}$, the pointwise Varadhan short-time asymptotic for the heat semigroup with respect to the Hausdorff distance induced by ${\mathsf {d}}$, Corollary 4.10;
- the irreducibility of the Dirichlet form $\mathsf {Ch}_{}$, Corollary 4.6.

For these results, we make full use of the fundamental relations between Dirichlet forms and metric measure spaces developed in [9].

Regarding the irreducibility, we note that the same proof of Corollary 4.6 applies as well to ${\mathsf {RCD}}(K,\infty )$ spaces with infinite volume measure, which seems not explicitly proved in the existing literature; see Remark 4.7 for a more detailed discussion.

Our results on ${\mathsf {QCD}}$ spaces may be specialized to ideal sub-Riemannian manifolds satisfying the quasi curvature-dimension condition, such as: (ideal generalized *H*-type) Carnot groups, Heisenberg groups, corank-1 Carnot groups, the Grushin plane, and several *H*-type foliations, Sasakian and 3-Sasakian manifolds.

## Milman’s quasi curvature-dimension-condition

By a *metric measure space* $(X,{\mathsf {d}},{\mathsf {m}})$ we shall always mean a complete and separable metric space $(X,{\mathsf {d}})$, endowed with a Borel measure ${\mathsf {m}}$ finite on ${\mathsf {d}}$-bounded sets and with full topological support. In order to rule out trivial cases, we assume that ${\mathsf {m}}$ is atomless, which makes *X* uncountable. We say that $(X,{\mathsf {d}})$ is *proper* if all closed balls are compact. We let ${\mathcal {C}}_c(X)$, resp. ${\mathcal {C}}_0(X)$, ${\mathcal {C}}_b(X)$, be the space of continuous compactly supported, resp. continuous vanishing at infinity, continuous bounded, functions on *X*.

We denote by ${\mathscr {P}}(X)$, resp. ${\mathscr {P}}_c(X)$, ${\mathscr {P}}^{\mathsf {m}}(X)$, the space of all Borel probability measures on $(X,{\mathsf {d}})$, resp. (additionally) compactly supported, (additionally) absolutely continuous w.r.t. ${\mathsf {m}}$, and by

$$\begin{aligned} {\mathscr {P}}_2(X):=\left\{ \mu \in {\mathscr {P}}(X): \int \limits _X {\mathsf {d}}(x,x_0)^2\mathop {}\!\mathrm {d}\mu <\infty \right\} \end{aligned}$$

the $L^2$-*Wasserstein space* over $(X,{\mathsf {d}})$, endowed with the $L^2$-*Wasserstein distance*

2.1 $$\begin{aligned} W_2(\mu _0,\mu _1):=\left[ \inf _\pi \int \limits _{X^{\scriptscriptstyle {\times 2}}} {\mathsf {d}}(x,y)^2\mathop {}\!\mathrm {d}\pi (x,y)\right] ^{1/2}, \end{aligned}$$

the infimum running over all couplings $\pi \in {\mathscr {P}}(X^{\scriptscriptstyle {\times 2}})$ of $(\mu _0,\mu _1)$. We denote by ${\mathrm {Opt}}(\mu _0,\mu _1)$ the set of minimizers in (2.1), always non-empty.

Set $I:=[0,1]$. We write ${\mathrm {Geo}}(X,{\mathsf {d}})$ for the space of all constant-speed geodesics in $(X,{\mathsf {d}})$ parametrized on *I*, itself a complete separable metric space when endowed with the supremum distance ${\mathsf {d}}_\infty$ induced by ${\mathsf {d}}$. By Lisini’s *superposition principle* [18, Thm. 4] (cf. [1, Thm. 2.10]), every $W_2$-absolutely continuous curve $\left( \mu _t\right) _{t\in I}$ may be lifted to a dynamical plan ${\varvec{\pi }}\in {\mathscr {P}}({\mathcal {C}}(I;X))$ satisfying $({\mathrm {ev}}_t)_\sharp {\varvec{\pi }}=\mu _t$ for every $t\in I$, where ${\mathrm {ev}}_t:\gamma \mapsto \gamma _t$ is the evaluation map at time *t*. Furthermore, a curve $\left( \mu _t\right) _t$ is a $W_2$-geodesic if and only if ${\varvec{\pi }}$ is concentrated on ${\mathrm {Geo}}(X,{\mathsf {d}})$ and

2.2 $$\begin{aligned} W_2(\mu _0,\mu _1)^2=\int \limits _{{\mathcal {C}}(I;X)} \int \limits _I \left|{{\dot{\gamma }}}\right|_t^2\mathop {}\!\mathrm {d}t \mathop {}\!\mathrm {d}{\varvec{\pi }}(\gamma )\mathrm {,}\end{aligned}$$

in which case we say that ${\varvec{\pi }}$ is an *optimal dynamical plan connecting* $\mu _0$ *and* $\mu _1$. We write ${\mathrm {OptGeo}}(\mu _0,\mu _1)$ for the set of all such plans.

Whenever $(Y,\tau )$ is a Polish space, the narrow topology $\tau _{\mathrm {n}}$ on the space ${\mathscr {P}}(Y)$ of Borel probability measures on *Y* is defined as the topology induced by duality with continuous bounded functions on *Y*. Since $(Y,\tau )$ is Polish, $({\mathscr {P}}(Y),\tau _{\mathrm {n}})$ is Polish as well, $\tau _{\mathrm {n}}$ is characterized by the convergence of sequences, and a sequence $\left( \mu _n\right) _n$ converges narrowly if and only if

$$\begin{aligned} \int \limits _X f\mathop {}\!\mathrm {d}\mu _n \xrightarrow {\ n\rightarrow \infty \ } \int \limits _X f \mathop {}\!\mathrm {d}\mu \mathrm {,}\quad f\in {\mathcal {C}}_b(X). \end{aligned}$$

We collect here for further reference the following standard fact.

### Proposition 2.1

(Stability of dynamical optimality). For $i=0,1$ let $\left( \mu ^n_i\right) _n\subset {\mathscr {P}}_2(X)$ and fix ${\varvec{\pi }}^n\in {\mathrm {OptGeo}}(\mu ^n_0,\mu ^n_1)$. If ${\varvec{\pi }}^n$ narrowly converges to ${\varvec{\pi }}\in {\mathscr {P}}({\mathcal {C}}(I;X))$, then ${\varvec{\pi }}$ is concentrated on ${\mathrm {Geo}}(X,{\mathsf {d}})$, and ${\varvec{\pi }}\in {\mathrm {OptGeo}}\big ({({\mathrm {ev}}_0)_\sharp {\varvec{\pi }}, ({\mathrm {ev}}_1)_\sharp {\varvec{\pi }}}\big )$.

### Proof

Consequence of the stability of $W_2$-optimality [1, Prop. 2.5], the continuity of $({\mathrm {ev}}_0,{\mathrm {ev}}_1):{\mathrm {Geo}}(X,{\mathsf {d}})\rightarrow X^{\scriptscriptstyle {\times 2}}$, and the Continuous Mapping Theorem. $\square$

### Definition 2.2

(*Monge space,*  [20, Dfn. 3.1]). A metric measure space $(X,{\mathsf {d}},{\mathsf {m}})$ is a *Monge space* if for every $\mu _0,\mu _1\in {\mathscr {P}}_2(X)$ with $\mu _0\ll {\mathsf {m}}$ the following holds:

- there exists a unique optimal dynamical plan ${\varvec{\pi }}\in {\mathrm {OptGeo}}(\mu _0,\mu _1)$, hence ${\mathrm {Opt}}(\mu _0,\mu _1)$ consists of the unique optimal plan $({\mathrm {ev}}_0,{\mathrm {ev}}_1)_\sharp {\varvec{\pi }}$;
- $(X,{\mathsf {d}},{\mathsf {m}})$ has *good transport behavior* (cf. [16, Dfn. 3.1]), i.e. ${\varvec{\pi }}$ is induced by a map, viz. ${\varvec{\pi }}={\mathbf {T}}_\sharp \mu _0$ for some ${\mathbf {T}}:X\rightarrow {\mathrm {Geo}}(X,{\mathsf {d}})$;
- $(X,{\mathsf {d}},{\mathsf {m}})$ has the *strong interpolation property* [16, p. 523], i.e. the optimal dynamical plan ${\varvec{\pi }}$ in (b) satisfies $\mu _t:=({\mathrm {ev}}_t)_\sharp {\varvec{\pi }}\ll {\mathsf {m}}$ for all $t\in [0,1)$.

We write $\rho _t$ for the Radon–Nikodým density of $\mu _t$ w.r.t. ${\mathsf {m}}$.

Let us now collect some properties of Monge spaces.

### Remark 2.3

Every geodesic Monge space is (2-)*essentially non-branching* [16, Dfn. 2.10] by [16, Prop. 3.6].

### Corollary 2.4

Let $(X,{\mathsf {d}},{\mathsf {m}})$ be a geodesic Monge space and fix $K_i\Subset X$, $i=0,1$. Then, the map

2.3 $$\begin{aligned} \Pi :(\mu _0,\mu _1)\longmapsto {\varvec{\pi }}\in {\mathrm {OptGeo}}(\mu _0,\mu _1) \end{aligned}$$

is a continuous map ${\mathscr {P}}^{\mathsf {m}}(K_0)\times {\mathscr {P}}(K_1)\rightarrow {\mathscr {P}}({\mathrm {Geo}}(X,{\mathsf {d}}))$ when ${\mathscr {P}}^{\mathsf {m}}(K_0)\times {\mathscr {P}}(K_1)$ is endowed with the product of the narrow topologies, and ${\mathscr {P}}({\mathrm {Geo}}(X,{\mathsf {d}}))$ is endowed with the narrow topology.

### Proof

Firstly, note that $\Pi$ is well-defined by Definition 2.2(a). Secondly, recall that the space of geodesics

$$\begin{aligned} {\mathrm {Geo}}(K_0,K_1):=\left\{ \gamma \in {\mathrm {Geo}}(X,{\mathsf {d}}): {\mathrm {ev}}_i \gamma \in K_i\right\} \end{aligned}$$

is a compact subset of ${\mathrm {Geo}}(X,{\mathsf {d}})$. As a consequence, ${\mathscr {P}}({\mathrm {Geo}}(K_0,K_1))$ is narrowly compact metrizable, and it suffices to show the continuity of $\Pi$ along sequences. To this end let $\left( \mu ^n_0\right) _n\subset {\mathscr {P}}^{\mathsf {m}}(K_0)$, and $\left( \mu ^n_1\right) _n\subset {\mathscr {P}}(K_1)$, be narrowly convergent to $\mu _0\in {\mathscr {P}}^{\mathsf {m}}(K_0)$, resp. $\mu _1\in {\mathscr {P}}(K_1)$. Since $\left( \Pi (\mu ^n_0,\mu ^n_1)\right) _n\subset {\mathscr {P}}({\mathrm {Geo}}(K_0,K_1))$, it admits a non-relabeled narrowly convergent subsequence. Let ${\varvec{\pi }}$ be its limit, and note that ${\varvec{\pi }}\in {\mathscr {P}}({\mathrm {Geo}}(X,{\mathsf {d}}))$ is an optimal dynamical plan by Proposition 2.1. By continuity of $({\mathrm {ev}}_0,{\mathrm {ev}}_1):{\mathrm {Geo}}(X,{\mathsf {d}})\rightarrow X^{\scriptscriptstyle {\times 2}}$, the narrow convergence of ${\varvec{\pi }}^n$ to ${\varvec{\pi }}$, and the Continuous Mapping Theorem, we conclude that ${\varvec{\pi }}\in {\mathrm {OptGeo}}(\mu _0,\mu _1)$. Since the latter is a singleton by Definition 2.2(a), we have therefore ${\varvec{\pi }}=\Pi (\mu _0,\mu _1)$. Since the subsequence was arbitrary, we have concluded that $\lim _{n}\Pi (\mu ^n_0,\mu ^n_1)=\lim _{n}{\varvec{\pi }}^n={\varvec{\pi }}=\Pi (\mu _0,\mu _1)$, which proves the assertion. $\square$

The following is a consequence of the sole *interpolation property* [16, Dfn. 4.2].

### Corollary 2.5

Let $(X,{\mathsf {d}},{\mathsf {m}})$ be a geodesic Monge space. Then, every ball $B\subset X$ is a continuity set for ${\mathsf {m}}$, i.e. ${\mathsf {m}}\, \partial B=0$. In particular, the sphere

$$\begin{aligned} S_r(x):=\left\{ y\in X: {\mathsf {d}}(x,y)=r\right\} \end{aligned}$$

is ${\mathsf {m}}$-negligible for every $x\in X$ and every $r>0$.

### Proof

Since $(X,{\mathsf {d}},{\mathsf {m}})$ has the strong interpolation property (Dfn. 2.2(c)), it has in particular the interpolation property, and is therefore *strongly non-degenerate* [16, Dfn. 4.4] by [16, Lem. 4.5]. In particular, it is *non-degenerate*, i.e., for every Borel $A\subset X$ with ${\mathsf {m}}A>0$ and every $x\in X$ it holds that ${\mathsf {m}}A_{t,x}>0$ for every $t\in (0,1)$, where

$$\begin{aligned} A_{t,x}:=\left\{ \gamma _t : \gamma \in {\mathrm {Geo}}(X,{\mathsf {d}})\mathrm {,}\gamma _0\in A, \gamma _1=x\right\} . \end{aligned}$$

Now, argue by contradiction that there exist $x_0\in X$ and $r_0>0$ with ${\mathsf {m}}S_{r_0}(x_0)>0$. On the one hand, since ${\mathsf {m}}$ is $\sigma$-finite we can find $r\in (0,r_0)$ with ${\mathsf {m}}S_r(x_0)=0$. On the other hand, since $S_{r}(x_0)=\big ({S_{r_0}(x_0)}\big )_{t,x_0}$ for $t:=r/r_0\in (0,1)$, the non-degeneracy implies ${\mathsf {m}}S_{r}(x_0)>0$, a contradiction.

Thus, every sphere $S_r(x)\subset X$ is ${\mathsf {m}}$-negligible. Since $(X,{\mathsf {d}})$ is geodesic, $S_r(x)=\partial B_r(x)$, and the first assertion follows. $\square$

The following generalization of Lott–Sturm–Villani curvature-dimension condition was recently introduced by Milman [20]. For $K\in {{\mathbb {R}}}$, $N\in (1,\infty )$ and $t\in (0,1)$, denote by $\tau ^{\scriptscriptstyle {(t)}}_{K,N}$ the *dynamical distortion coefficient* of the model space of constant sectional curvature $\frac{K}{N-1}$ and dimension $\left\lceil N\right\rceil$, e.g. [20, Eq. (2.2)].

### Definition 2.6

(*Quasi Curvature-Dimension Condition,* [20, Dfn.s 2.3,2.8]). For $Q\ge 1$, $K\in {{\mathbb {R}}}$, and $N\in (1,\infty )$, a geodesic Monge space $(X,{\mathsf {d}},{\mathsf {m}})$ satisfies the *quasi curvature-dimension condition* ${\mathsf {QCD}}(Q,K,N)$ if, for every $\mu _0,\mu _1\in {\mathscr {P}}^{\mathsf {m}}_c(X)$,

2.4 $$\begin{aligned} \rho _t^{-1/N}(\gamma _t) \ge Q^{-1/N} \left( \tau ^{\scriptscriptstyle {(1-t)}}_{K,N}\big ({{\mathsf {d}}(\gamma _0,\gamma _1)}\big )\, \rho _0^{-1/N}(\gamma _0)+ \tau ^{\scriptscriptstyle {(t)}}_{K,N}\big ({{\mathsf {d}}(\gamma _0,\gamma _1)}\big )\, \rho _1^{-1/N}(\gamma _1)\right) \end{aligned}$$

for every $t\in (0,1)$ and ${\varvec{\pi }}$-a.e. $\gamma \in {\mathrm {Geo}}(X,{\mathsf {d}})$.

We further say that $(X,{\mathsf {d}},{\mathsf {m}})$ satisfies the *regular quasi curvature-dimension condition* ${\mathsf {QCD}}_{\mathrm {reg}}(Q,K,N)$ if it satisfies ${\mathsf {QCD}}(Q,K,N)$ for some *Q*, *K*, *N* as above and additionally the *measure contraction property* ${\mathsf {MCP}}(K',N')$ for some $K'\in {{\mathbb {R}}}$ and $N'\in (1,\infty )$.

In the following, we omit the indices *Q*, *K*, and *N* whenever not relevant. We refer to [20] for a thorough discussion of examples of spaces satisfying the ${\mathsf {QCD}}$ condition. We stress that they include all ${\mathsf {CD}}$ spaces (for the choice $Q=1$, see [20]), and various classes of sub-Riemannian manifolds satisfying ${\mathsf {MCP}}$ (see [20, Prop. 2.4]).

Since the right-hand side of (2.4) depends on *Q* only by its linear dependence on the constant $Q^{-1/N}$, the proof of the following result is readily adapted from the one of the analogous assertion under the curvature-dimension condition ${\mathsf {CD}}$ in [29, Thm. 2.3].

For fixed $x_0\in X$ and for every $r>0$, set ${\overline{B}}_r(x_0):=\left\{ x\in X: {\mathsf {d}}(x,x_0)\le r\right\}$, and

$$\begin{aligned} v(r):={\mathsf {m}}B_r(x_0) \quad \text {and} \quad s(r):=\limsup _{\delta \downarrow 0} \frac{1}{\delta } \, {\mathsf {m}}\big ({{\overline{B}}_{r+\delta }(x_0)\setminus B_r(x_0)}\big ). \end{aligned}$$

As customary, further define the model volume coefficient

$$\begin{aligned} s_{K,N}(r):={\left\{ \begin{array}{ll} \sin \left( \sqrt{\tfrac{K}{N-1}} \, r\right) &{} \text {if } K>0\\ r &{} \text {if } K=0\\ \sinh \left( \sqrt{\tfrac{-K}{N-1}} \, r\right) &{} \text {if } K<0 \end{array}\right. }. \end{aligned}$$

### Lemma 2.7

(Generalized Bishop–Gromov inequality). Let $(X,{\mathsf {d}},{\mathsf {m}})$ be a metric measure space satisfying ${\mathsf {QCD}}(Q,K,N)$ for some $Q\ge 1$, $K\in {{\mathbb {R}}}$, and $N\in (1,\infty )$. Then, for every $0<r\le R$ *(*with $R\le \pi /\sqrt{K/(N-1)}$ if $K>0$*)*,

2.5 $$\begin{aligned} \frac{s(r)}{s(R)}&\ge Q^{-N}\, \left( \frac{s_{K,N}(r)}{s_{K,N}(R)}\right) ^{N-1}\mathrm {,}\end{aligned}$$

2.6 $$\begin{aligned} \frac{v(r)}{v(R)}&\ge Q^{-N}\, \frac{\int _0^r s_{K,N}(t)^{N-1}\mathop {}\!\mathrm {d}t}{\int _0^R s_{K,N}(t)^{N-1}\mathop {}\!\mathrm {d}t}. \end{aligned}$$

### Remark

If $Q>1$, then (2.6) is not sufficient to conclude that $v(r)/v(R)\rightarrow 1$ as $r\rightarrow R$. In particular, this implies that the assertion of Corollary 2.5 does not follow from (2.6) in the obvious way, which makes the Corollary non-void.

### Proof of Lemma 2.7

Let $A_0,A_1$ be Borel subsets of *X* with ${\mathsf {m}}A_0, {\mathsf {m}}A_1>0$, and set

$$\begin{aligned} A_{t}:=\left\{ \gamma _t : \gamma \in {\mathrm {Geo}}(X,{\mathsf {d}})\mathrm {,}\gamma _i\in A_i\mathrm {,}i=0,1\right\} \mathrm {.}\end{aligned}$$

Following *verbatim* the proof of [29, Prop. 2.1] yields the following *Q*-weighted version of the generalized Brunn–Minkowski inequality:

2.7 $$\begin{aligned} ({\mathsf {m}}A_t)^{1/N}\ge Q^{-1} \left( \tau ^{{\scriptscriptstyle {(1-t)}}}_{K,N} (\Theta )\, ({\mathsf {m}}A_0)^{1/N}+ \tau ^{{\scriptscriptstyle {(t)}}}_{K,N} (\Theta )\, ({\mathsf {m}}A_1)^{1/N}\right) \mathrm {,}\end{aligned}$$

where

$$\begin{aligned} \Theta :={\left\{ \begin{array}{ll} \inf _{x_i\in A_i} {\mathsf {d}}(x_0,x_1) &{} \text {if } K\ge 0\\ \sup _{x_i\in A_i}{\mathsf {d}}(x_0,x_1) &{}\text {if } K<0\end{array}\right. } \mathrm {.}\end{aligned}$$

We apply (2.7) to $A_0:=B_\varepsilon (x_0)$ and $A_1:={\overline{B}}_{R+\delta R}(x_0)\setminus B_R(x_0)$ for some $\varepsilon ,\delta >0$, fixed $0<r<R$, and with $t:=r/R$. It is readily seen that

$$\begin{aligned} A_t\subset {\overline{B}}_{r+\delta r+\varepsilon r/R}(x_0)\setminus B_{r-\varepsilon r/R}(x_0) \qquad \text {and} \qquad R-\varepsilon \le \Theta \le R+\delta R+\varepsilon \mathrm {.}\end{aligned}$$

Thus, by (2.7),

$$\begin{aligned}&{\mathsf {m}}\big ({{\overline{B}}_{r+\delta r+\varepsilon r/R}(x_0)\setminus B_{r-\varepsilon r/R}(x_0)}\big )^{1/N}\\&\quad \ge Q^{-1} \tau ^{\scriptscriptstyle {(1-r/R)}}_{K,N} (R\mp \delta R \mp \varepsilon ) \big ({{\mathsf {m}}B_\varepsilon (x_0)}\big )^{1/N}\\&\qquad +Q^{-1}\tau ^{{\scriptscriptstyle {(r/R)}}}_{K,N}(R\mp \delta R\mp \varepsilon )\, {\mathsf {m}}\big ({{\overline{B}}_{R+\delta R}(x_0)\setminus B_R(x_0)}\big )^{1/N} \end{aligned}$$

where $\mp$ is chosen to coincide with ${\mathrm {sgn}}(K)$. Letting $\varepsilon \rightarrow 0$,

$$\begin{aligned} {\mathsf {m}}&\big ({{\overline{B}}_{(1+\delta )r}(x_0)\setminus B_r(x_0)}\big )^{1/N}\ge Q^{-1}\tau ^{{\scriptscriptstyle {(r/R)}}}_{K,N}\big ({(1\mp \delta ) R}\big )\, {\mathsf {m}}\big ({{\overline{B}}_{R+\delta R}(x_0)\setminus B_R(x_0)}\big )^{1/N} \mathrm {.}\end{aligned}$$

Since ${\mathsf {m}}$ does not charge spheres by Corollary 2.5, we may rewrite the above inequality as

$$\begin{aligned} v\big ({(1+\delta )r}\big )-v(r) \ge Q^{-N}\, \tau ^{{\scriptscriptstyle {(r/R)}}}_{K,N}\big ({(1\mp \delta )R}\big )^N \left( v\big ({(1+\delta )R}\big )-v(R)\right) \mathrm {,}\end{aligned}$$

hence, making explicit the definition of the distortion coefficients,

2.8 $$\begin{aligned} \frac{v\big ({(1+\delta )r}\big )-v(r)}{\delta r}\ge Q^{-N} \frac{v\big ({(1+\delta )R}\big )-v(R)}{\delta R} \left( \frac{s_{K,N}\big ({(1\mp \delta )r}\big )}{s_{K,N}\big ({(1\mp \delta )R}\big )}\right) ^{N-1}. \end{aligned}$$

Letting $\delta \rightarrow 0$ in (2.8) proves (2.5). The inequality (2.6) now follows from (2.5) exactly as in the proof of [29, Thm. 2.3]. $\square$

Recall that a metric measure space $(X,{\mathsf {d}},{\mathsf {m}})$ is *locally doubling* if for every $x\in X$ there exists an open set $U\ni x$ and constants $C,R>0$ so that

$$\begin{aligned} {\mathsf {m}}B_{2r}(y)\le C {\mathsf {m}}B_r(y)\mathrm {,}\qquad r\in (0,R)\mathrm {,}\qquad y\in U\mathrm {.}\end{aligned}$$

Following [13, Dfn. 3.18], we further say that $(X,{\mathsf {d}},{\mathsf {m}})$ is *a.e.-locally doubling* if there exists an ${\mathsf {m}}$-negliglible set $N\subset X$ so that $X\setminus N$ is locally doubling when endowed with the restriction of ${\mathsf {d}}$ and ${\mathsf {m}}$.

As standard corollaries of Lemma 2.7, we further have the following properties:

### Corollary 2.8

Every ${\mathsf {QCD}}$ space is locally doubling.

### Corollary 2.9

Every ${\mathsf {QCD}}$ space is proper.

## The Sobolev-to-Lipschitz property on ${\mathsf {QCD}}$-spaces

Let $(X,{\mathsf {d}},{\mathsf {m}})$ be a metric measure space. We denote by $\mathrm {L}_{}(f)$ the global Lipschitz constant of a Lipschitz function $f:X\rightarrow {{\mathbb {R}}}$, and by ${\mathrm {Lip}}({\mathsf {d}})$, resp. ${\mathrm {Lip}}_{bs}({\mathsf {d}})$, the space of all Lipschitz functions, resp. (additionally) with bounded support, on $(X,{\mathsf {d}})$. We briefly recall the definition of Cheeger energy of a metric measure space. For a function $f\in {\mathrm {Lip}}({\mathsf {d}})$, define the slope of *f* at *x* by

$$\begin{aligned} \left|\mathrm {D}f\right|_{}(x):=\limsup _{y\rightarrow x} \frac{\left|f(y)-f(x)\right|}{{\mathsf {d}}(x,y)}\mathrm {,}\end{aligned}$$

where, conventionally, $\left|\mathrm {D}f\right|_{}(x)=0$ if *x* is isolated. The *Cheeger energy* [2, Eq. (4.11)] on $(X,{\mathsf {d}},{\mathsf {m}})$ is the functional

$$\begin{aligned} \mathsf {Ch}_{{\mathsf {d}},{\mathsf {m}}}(f):=\inf \left\{ \liminf _{n }\int \limits _{X} \left|\mathrm {D}f_n\right|_{}^2 \mathop {}\!\mathrm {d}{\mathsf {m}}: f_n\in {\mathrm {Lip}}_{bs}({\mathsf {d}})\mathrm {,}L^2({\mathsf {m}})\text {-}\lim _{n}f_n=f\right\} \mathrm {,}\end{aligned}$$

where, conventionally, $\inf {{\,\mathrm{\varnothing }\,}}:=+\infty$. We denote the domain of $\mathsf {Ch}_{{\mathsf {d}},{\mathsf {m}}}$ by

$$\begin{aligned} W^{1,2}= W^{1,2}(X, {\mathsf {d}}. {\mathsf {m}}):=\left\{ f \in L^2(X, {\mathsf {m}}): \mathsf {Ch}_{{\mathsf {d}},{\mathsf {m}}}(f)<\infty \right\} \mathrm {.}\end{aligned}$$

We recall that a metric measure space $(X,{\mathsf {d}},{\mathsf {m}})$ is called *infinitesimally Hilbertian* if $\mathsf {Ch}_{{\mathsf {d}},{\mathsf {m}}}$ is quadratic. Introduced by Gigli in [12, Dfn. 4.19], this notion has ever since proven to be a key tool in the study of non-smooth metric measure spaces.

When $(X,{\mathsf {d}},{\mathsf {m}})$ is infinitesimally Hilbertian, $\mathsf {Ch}_{{\mathsf {d}},{\mathsf {m}}}$ is a strongly local Dirichlet form having square-field operator $\left|\mathrm {D}f\right|_{w}^2$, where $\left|\mathrm {D}f\right|_{w}$ is called the weak minimal upper gradient, satisfying—by construction—the *Rademacher-type property*: 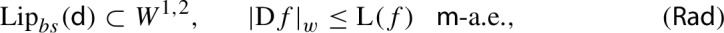 where ${\mathrm {Lip}}_{bs}$ denotes the space of Lipschitz functions with bounded support.

The following property has been considered in a variety of non-smooth settings, including e.g. configuration spaces [25], or general metric measure spaces [11, Dfn. 4.9].

### Definition 3.1

(*Sobolev-to-Lipschitz property*). We say that a metric measure space $(X,{\mathsf {d}},{\mathsf {m}})$ satisfies the *Sobolev-to-Lipschitz property* if 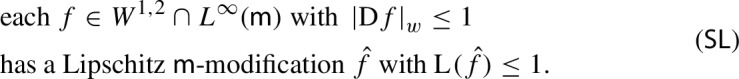


### Remark 3.2

The Sobolev-to-Lipschitz property is more commonly phrased without the requirement that $f\in L^\infty ({\mathsf {m}})$. In fact, this is equivalent to .

### Proof

Let $f\in W^{1,2}$ with $\left|\mathrm {D}f\right|_{w}\le 1$, and set $f_r:=-r \vee f \wedge r$ for each $r>0$. By locality of $\left|\mathrm {D}{\,\cdot \,}\right|_{w}$ we have that $\left|\mathrm {D}f_r\right|_{w}\le 1$ ${\mathsf {m}}$-a.e. for every *r*, hence $f_r$ has a Lipschitz ${\mathsf {m}}$-modification $\hat{f}_r$ with $\mathrm {L}_{}(\hat{f}_r)\le 1$, by . Since $\hat{f}_r$ is continuous and ${\mathsf {m}}$ has full support, we have that $\hat{f}_r\equiv \hat{f}_s$ everywhere on $\big \{x\in X:|\hat{f}_s(x)|\le r\big \}$ for every $s\ge r$. We conclude letting $r\rightarrow \infty$. $\square$

### Remark 3.3

(*On the Rademacher-type and Sobolev-to-Lipschitz properties*). As anticipated above, the Rademacher-type property for $\mathsf {Ch}_{{\mathsf {d}},{\mathsf {m}}}$ holds *by construction* on *every* metric measure space (even without the assumption of infinitesimal Hilbertianity). However the Rademacher-type property becomes non-trivial in the general setting of strongly local Dirichlet forms, i.e. when $\mathsf {Ch}_{{\mathsf {d}},{\mathsf {m}}}$ is replaced by any such Dirichlet form ${\mathcal {E}}$ on $L^2({\mathsf {m}})$. In this case, the Sobolev-to-Lipschitz property and the Rademacher-type property may be regarded as converse to each other. For many comments and examples on both properties in this setting, see e.g. [9, Sects. 3–4].

Concerning our terminology, we ought to stress that the Rademacher-type property $(\mathsf {Rad})$ we defined here does not entail any (strong) differentiability nor any Gâteaux (i.e. directional) differentiability of the function involved. Indeed, even phrasing any such concept of (Gâteaux) differentiability on general metric measure spaces would be highly non-trivial. While immediate on metric measure spaces (as discussed above), the property $(\mathsf {Rad})$ is non-trivial on general Dirichlet spaces when $\left|\mathrm {D}{\,\cdot \,}\right|_{}$ is replaced by the square field operator (or even by the energy measure) of a strongly local Dirichlet form, see e.g. [9, 17, 27]. In this case, proofs of strong notions of differentiability of Lipschitz functions are available in specific smooth and non-smooth settings, which invariably rely on a combination of Gâteaux differentiability along ‘sufficiently many’ directions together with the uniform bound on such derivatives, provided by $(\mathsf {Rad})$; see e.g. [21] for Euclidean spaces, [6, 10] for Wiener and Banach spaces, [25] for configuration spaces, [7] for Wasserstein spaces, etc.

In order to discuss the Sobolev-to-Lipschitz property on ${\mathsf {QCD}}$ spaces, we recall the following definition by Gigli and Han [13]. Firstly, recall from [2, Dfn. 5.1] that a dynamical plan ${\varvec{\pi }}\in {\mathscr {P}}({\mathcal {C}}(I;X))$ is a *test plan* if

- it is concentrated on the family ${\mathrm {AC}}^2(I;X)$ of 2-absolutely continuous curves;
- it has finite 2-energy, i.e. the right-hand side of (2.2) is finite;
- it has *bounded compression*, viz. $({\mathrm {ev}}_t)_\sharp {\varvec{\pi }}\le C{\mathsf {m}}$ for some contant $C>0$ independent of $t\in I$.

### Definition 3.4

(*Measured-length space*, [13, Dfn. 3.16]). A metric measure space $(X,{\mathsf {d}}, {\mathsf {m}})$ is a *measured-length space* if there exists an ${\mathsf {m}}$-co-negligible subset $\Omega \subset X$ with the following property. For $i=0,1$ and every $x_i\in \Omega$ there exists $\varepsilon :=\varepsilon (x_0,x_1)>0$ such that for each $\varepsilon _i\in (0,\varepsilon ]$ there exists a test plan ${\varvec{\pi }}^{\varepsilon _0,\varepsilon _1}\in {\mathscr {P}}({\mathcal {C}}(I, X))$ such that:

- the map
  3.1 $$\begin{aligned} (0,\varepsilon ]^{\scriptscriptstyle {\times 2}}\ni (\varepsilon _0,\varepsilon _1)\longmapsto {\varvec{\pi }}^{\varepsilon _0,\varepsilon _1} \end{aligned}$$
  is weakly Borel measurable, viz.
  $$\begin{aligned} (\varepsilon _0,\varepsilon _1)\longmapsto \int \varphi \mathop {}\!\mathrm {d}{\varvec{\pi }}^{\varepsilon _0,\varepsilon _1} ~\text {is Borel measurable}~\qquad \varphi \in {\mathcal {C}}_b({\mathcal {C}}(I,X)); \end{aligned}$$
- letting ${\mathrm {ev}}_t:{\mathcal {C}}(I,M)\ni \gamma \mapsto \gamma _t\in M$ be the evaluation map on curves, it holds that
  $$\begin{aligned} ({\mathrm {ev}}_i)_\sharp {\varvec{\pi }}^{\varepsilon _0,\varepsilon _1}=\frac{\mathbb {1}_{B_{\varepsilon _i}(x_i)}}{{\mathsf {m}}B_{\varepsilon _i(x_i)}},\quad \varepsilon _i\in (0,\varepsilon ]; \end{aligned}$$
- we have that
  $$\begin{aligned} \limsup _{\varepsilon _0,\varepsilon _1\downarrow 0} \iint \limits _I \left|{{\dot{\gamma }}}\right|^2 \mathop {}\!\mathrm {d}t \mathop {}\!\mathrm {d}{\varvec{\pi }}^{\varepsilon _0,\varepsilon _1}(\gamma ) \le {\mathsf {d}}(x_0,x_1)^2. \end{aligned}$$

### Proposition 3.5

([13, Prop. 3.19]). Every a.e.-locally doubling measured-length space satisfies .

### Theorem 3.6

Every ${\mathsf {QCD}}$ space is a measured-length space.

### Proof

Let $x_i\in X$, $i=0,1$, and assume $x_0\ne x_1$.

*Definition of* ${\varvec{\pi }}$ Set $\varepsilon :={\mathsf {d}}(x_0,x_1)/4>0$ and, for $\varepsilon _i<\varepsilon$, let

3.2 $$\begin{aligned} \mu ^{\varepsilon _i}:=\frac{\mathbb {1}_{B_{\varepsilon _i}(x_i)}}{{\mathsf {m}}B_{\varepsilon _i}(x_i)}\cdot {\mathsf {m}}\mathrm {,}\end{aligned}$$

and

$$\begin{aligned} G^{\varepsilon _0,\varepsilon _1}:=\left\{ \gamma \in {\mathrm {Geo}}(X,{\mathsf {d}}), \gamma _i\in B_{\varepsilon _i}(x_i), i=0,1\right\} . \end{aligned}$$

By definition of $\varepsilon _0,\varepsilon _1,\varepsilon$, the sets ${\mathrm {supp}}\mu ^{\varepsilon _0}$ and ${\mathrm {supp}}\mu ^{\varepsilon _1}$ are well-separated, and compact since $(X,{\mathsf {d}})$ is proper. Therefore, by Definition 2.2(a), there exists an optimal dynamical plan ${\varvec{\pi }}^{\varepsilon _0,\varepsilon _1}\in {\mathrm {OptGeo}}(\mu ^{\varepsilon _0},\mu ^{\varepsilon _1})$.

*Properties of* ${\varvec{\pi }}$ Note that ${\varvec{\pi }}^{\varepsilon _0,\varepsilon _1}$ is concentrated on $G^{\varepsilon _0,\varepsilon _1}$. By [18, Thm. 5], every optimal dynamical plan is concentrated on ${\mathrm {AC}}^2(I;X)$ and has finite 2-energy. Therefore, an optimal dynamical plan is a test plan if and only if it has bounded compression. Let us show that ${\varvec{\pi }}^{\varepsilon _0,\varepsilon _1}$ has bounded compression. For $t\in I$ let $\mu ^{\varepsilon _0,\varepsilon _1}_t:=({\mathrm {ev}}_t)_\sharp {\varvec{\pi }}^{\varepsilon _0,\varepsilon _1}=\rho _t{\mathsf {m}}$ be the $W_2$-geodesic connecting $\mu ^{\varepsilon _0}$ to $\mu ^{\varepsilon _1}$. By the quasi curvature-dimension condition, (2.4) holds for some $Q\ge 1$, $K\in {{\mathbb {R}}}$, and $N\in (1,\infty )$. As a consequence, for ${\varvec{\pi }}^{\varepsilon _0,\varepsilon _1}$-a.e. $\gamma$,

$$\begin{aligned} \rho _t(\gamma _t)&\le Q \left( \tau ^{\scriptscriptstyle {(1-t)}}_{K,N}\big ({{\mathsf {d}}(\gamma _0,\gamma _1)}\big )\, \rho _0^{-1/N}(\gamma _0)+ \tau ^{\scriptscriptstyle {(t)}}_{K,N}\big ({{\mathsf {d}}(\gamma _0,\gamma _1)}\big )\, \rho _1^{-1/N}(\gamma _1)\right) ^{-N}\\&= Q \left( \tau ^{\scriptscriptstyle {(1-t)}}_{K,N}\big ({{\mathsf {d}}(\gamma _0,\gamma _1)}\big )\, {\mathsf {m}}B_{\varepsilon _0}(x_0)^{1/N}+ \tau ^{\scriptscriptstyle {(t)}}_{K,N}\big ({{\mathsf {d}}(\gamma _0,\gamma _1)}\big )\, {\mathsf {m}}B_{\varepsilon _1}(x_1)^{1/N} \right) ^{-N} \\&\le Q\,\min \left\{ {\mathsf {m}}B_{\varepsilon _0}(x_0),{\mathsf {m}}B_{\varepsilon _1}(x_1)\right\} ^{-1} \left( \tau ^{\scriptscriptstyle {(1-t)}}_{K,N}\big ({{\mathsf {d}}(\gamma _0,\gamma _1)}\big )+ \tau ^{\scriptscriptstyle {(t)}}_{K,N}\big ({{\mathsf {d}}(\gamma _0,\gamma _1)}\big ) \right) ^{-N} \end{aligned}$$

which is finite uniformly in $t\in I$ since $\tau ^{\scriptscriptstyle {(t)}}_{K,N}(\theta )+\tau ^{\scriptscriptstyle {(1-t)}}_{K,N}(\theta )$ is bounded away from 0 uniformly in $t\in I$ locally uniformly in $\theta \in [0,\infty )$. Since $\rho _t$ is concentrated on ${\mathrm {ev}}_t(G^{\varepsilon _0,\varepsilon _1})$, the previous inequality concludes that ${\varvec{\pi }}^{\varepsilon _0,\varepsilon _1}$ has bounded compression.

In order to show Definition 3.4(c), note that, since ${\varvec{\pi }}^{\varepsilon _0,\varepsilon _1}\in {\mathrm {OptGeo}}(\mu ^{\varepsilon _0}, \mu ^{\varepsilon _1})$, then, in the constant speed parametrization,

$$\begin{aligned} \iint \limits _I \left|{{\dot{\gamma }}}\right|_t^2 \mathop {}\!\mathrm {d}t\mathop {}\!\mathrm {d}{\varvec{\pi }}^{\varepsilon _0,\varepsilon _1}(\gamma )\le \big ({{\mathsf {d}}(x_0,x_1)+\varepsilon _0+\varepsilon _1}\big )^2\xrightarrow {\ \varepsilon _0,\varepsilon _1\downarrow 0\ } {\mathsf {d}}(x_0,x_1)^2\mathrm {,}\end{aligned}$$

which proves the assertion.

It remains to show the measurability assertion in Definition 3.4(a). To this end, it suffices to note that, letting $E_i:\varepsilon _i\mapsto \mu ^{\varepsilon _i}$,

$$\begin{aligned} \Pi \circ (E_0, E_1):(\varepsilon _0,\varepsilon _1)\longmapsto {\varvec{\pi }}^{\varepsilon _0,\varepsilon _1} \end{aligned}$$

with $\Pi$ as in (2.3). Since $(X,{\mathsf {d}})$ is proper by Corollary 2.9, choosing $K_i:=B_\varepsilon (x_i)$ in Corollary 2.4 shows that the map $\Pi$ is narrowly/narrowly continuous (hence Borel) on the image of $(E_0, E_1)$. Thus, it suffices to show that $E_i$ is Borel for $i=0,1$. We show that $E_i$, $i=0,1$, is Euclidean/narrowly continuous. Let $f\in {\mathcal {C}}_b(X)$ be fixed. It suffices to show that $r\mapsto E_i(r) f$ is continuous on $[0,\varepsilon )$. The continuity at $r=0$ holds since ${\mathsf {m}}$ has no atoms. For $r,s>0$, we have that

$$\begin{aligned} \left|E_i(r)f-E_i(s)f\right|&\le \left|\frac{1}{{\mathsf {m}}B_r(x_i)}-\frac{1}{{\mathsf {m}}B_s(x_i)}\right| \int \limits _{B_r(x_i)} \left|f\right|\mathop {}\!\mathrm {d}{\mathsf {m}}\\&\quad + \frac{1}{{\mathsf {m}}B_s(x_i)} \int \limits _{B_r(x_i)\triangle B_s(x_i)}\left|f\right| \mathop {}\!\mathrm {d}{\mathsf {m}}. \end{aligned}$$

The second term vanishes as $r\rightarrow s$ by continuity of the measure ${\mathsf {m}}$. As for the first term, it suffices to show that $r\mapsto {\mathsf {m}}B_r(x_i)$ is continuous for $r\in [0,\varepsilon )$. This follows from the Portmanteau Theorem, since all balls in *X* are continuity sets for ${\mathsf {m}}$ by the first assertion in Corollary 2.5.

If $x_0=x_1$, then the requirements in Definition 3.4(b)–(c) hold trivially, and Definition 3.4(a) holds as in the case $x_0\ne x_1$ discussed above. $\square$

Combining Corollary 2.8 with Proposition 3.5 and Theorem 3.6, we conclude the Sobolev-to-Lipschitz property for ${\mathsf {QCD}}$ spaces.

### Corollary 3.7

Every ${\mathsf {QCD}}$ space satisfies .

### Remark 3.8

It would not be difficult to show that the original proof of  for ${\mathsf {CD}}(K,N)$ spaces [11, p. 48] can be adapted as well to the case of ${\mathsf {QCD}}(Q,K,N)$ spaces. The proof in [11] relies on an argument in [23] providing suitable test plans connecting the approximating measures $\mu ^{\varepsilon _i}$ in (3.2).

The proof we presented above makes instead use of the more refined notion of measured-length space in [13]. Whereas slightly more involved, this proof makes more explicit the relation between the inequality (2.4) defining the ${\mathsf {QCD}}$, and the upper bound on the compression for the aforementioned test plans.

## Properties of ${\mathsf {RQCD}}$ spaces

In this section, we prove several applications of  under the additional assumption that the Cheeger energy is quadratic.

### Definition 4.1

(*cf.* [20, Sect. 7.2]). Let $(X,{\mathsf {d}},{\mathsf {m}})$ be a metric measure space, $Q\ge 1$, $K\in {{\mathbb {R}}}$, and $N\in (1,\infty )$. We say it satisfies the *Riemannian quasi curvature-dimension condition* ${\mathsf {RQCD}}(Q,K,N)$ if it satisfies ${\mathsf {QCD}}(Q,K,N)$ and it is additionally infinitesimally Hilbertian, i.e. $\mathsf {Ch}_{{\mathsf {d}},{\mathsf {m}}}$ is a *quadratic* functional.

In the following, we omit the indices *Q*, *K*, and *N* whenever not relevant. Note that, when $(X,{\mathsf {d}},{\mathsf {m}})$ is an ${\mathsf {RQCD}}$ space, the quadratic form induced by the Cheeger energy of $(X,{\mathsf {d}},{\mathsf {m}})$ by polarization is a Dirichlet form, again denoted by $\mathsf {Ch}_{{\mathsf {d}},{\mathsf {m}}}$ and still called the Cheger energy of $(X,{\mathsf {d}},{\mathsf {m}})$.

Recall that a Dirichlet form $({\mathcal {E}},{\mathcal {F}})$ on a locally compact Polish space $(X,{\mathsf {d}},{\mathsf {m}})$ is called *regular* if ${\mathcal {F}}\cap {\mathcal {C}}_0(X)$ is both $\big ({{\mathcal {E}}({\,\cdot \,})+\left\Vert {\,\cdot \,}\right\Vert ^2_{L^2}}\big )^{1/2}$-dense in ${\mathcal {F}}$ and uniformly dense in ${\mathcal {C}}_0(X)$.

### Proposition 4.2

Let $(X,{\mathsf {d}},{\mathsf {m}})$ be an ${\mathsf {RQCD}}$ space. Then $(\mathsf {Ch}_{{\mathsf {d}},{\mathsf {m}}}, W^{1,2})$ is a regular strongly local Dirichlet form.

### Proof

The regularity follows directly from the uniform density of ${\mathrm {Lip}}_{bs}({\mathsf {d}})$ in ${\mathcal {C}}_0(X)$ and from the norm density of ${\mathrm {Lip}}_{bs}$ in $W^{1,2}$. The strong locality is then a standard consequence of the locality of the weak upper gradient $\left|\mathrm {D}{\,\cdot \,}\right|_{w}$. $\square$

### Example 4.3

(*sub-Riemannian manifolds*). Let $(M,{\mathcal {H}})$ be a sub-Riemannian manifold with smooth non-holonomic distribution ${\mathcal {H}}$ on *TM*.

On the one hand, by the Chow–Rashevskii Theorem, endowing *M* with its Carnot–Carathéodory distance ${\mathsf {d}}_{\textsc {cc}}$ and with a smooth measure ${\mathsf {m}}$ turns it into a proper metric measure space, thus admitting a Cheeger energy $\mathsf {Ch}_{{\mathsf {d}}_{\textsc {cc}},{\mathsf {m}}}$. On the other hand, the sub-Laplacian *L* induced by the distribution ${\mathcal {H}}$ generates a regular Dirichlet form $({\mathcal {E}},{\mathcal {F}})$ on $L^2({\mathsf {m}})$ with core ${\mathcal {C}}^\infty _c(X)$, defined by ${\mathcal {E}}(f,g)=\left\langle f \,|\, -Lg\right\rangle _{L^2({\mathsf {m}})}$, see e.g. [5, p. 191].

In fact, for a sub-Riemannian manifold $(M,{\mathsf {d}}_{\textsc {cc}},{\mathsf {m}})$ as above, the Dirichlet form $({\mathcal {E}},{\mathcal {F}})$ coincides with the Cheeger energy $(\mathsf {Ch}_{{\mathsf {d}}_{\textsc {cc}},{\mathsf {m}}}, W^{1,2})$ of $(M,{\mathsf {d}}_{\textsc {cc}},{\mathsf {m}})$, viz.

4.1 $$\begin{aligned} ({\mathcal {E}},{\mathcal {F}})=(\mathsf {Ch}_{{\mathsf {d}}_{\textsc {cc}},{\mathsf {m}}},W^{1,2}), \end{aligned}$$

as we show below.

As a consequence, every sub-Riemannian manifold $(M,{\mathsf {d}}_{\textsc {cc}},{\mathsf {m}})$ satisfying the ${\mathsf {QCD}}$ condition satisfies as well the ${\mathsf {RQCD}}$ condition with same parameters. See [20] for relevant families of examples of sub-Riemannian manifolds satisfying the ${\mathsf {QCD}}$ condition.

*Proof of* (4.1) On the one hand, by [2, Thm. 6.2], the square field $\left|\mathrm {D}f\right|_{w}$ of $f\in W^{1,2}$ coincides with the minimal 2-weak upper gradient of *f*. On the other hand, by [14, Thm. 11.7], the square field $\Gamma (f)$ of $f\in {\mathcal {F}}\cap {\mathcal {C}}(X)$ coincides as well with the minimal 2-weak upper gradient of *f*. As a consequence, ${\mathcal {E}}(f)=\mathsf {Ch}_{{\mathsf {d}}_{\textsc {cc}},{\mathsf {m}}}(f)$ for every $f\in W^{1,2}\cap {\mathcal {C}}(X)$ as well, and the conclusion follows since $W^{1,2}\cap {\mathcal {C}}_0(X)$ is a core for $(\mathsf {Ch}_{{\mathsf {d}}_{\textsc {cc}},{\mathsf {m}}}, W^{1,2})$ by regularity of the latter (Prop. 4.2) and since ${\mathcal {F}}\cap {\mathcal {C}}_0(X)$ is a core for $({\mathcal {E}},{\mathcal {F}})$ by definition. $\square$

For a *regular* Dirichlet form $({\mathcal {E}},{\mathcal {F}})$ on $L^2({\mathsf {m}})$, the *local domain* ${\mathcal {F}}_{\mathrm {loc}}$ of ${\mathcal {E}}$ is defined as the space of all functions $f\in L^0({\mathsf {m}})$ so that, for each relatively compact open $G\subset X$ there exists $f_G\in {\mathcal {F}}$ with $f\equiv f_G$ ${\mathsf {m}}$-a.e. on *G*. When $({\mathcal {E}},{\mathcal {F}})$ admits square field ${\mathcal {F}}\ni (f,g)\mapsto \Gamma (f,g)\in L^1({\mathsf {m}})$, the quadratic form $f\mapsto \Gamma (f):=\Gamma (f,f)$ naturally extends to the local domain ${\mathcal {F}}_{\mathrm {loc}}$, e.g. [27, Sect. 4.1.i].

Let us define the following local versions of the Rademacher-type and Sobolev-to-Lipschitz properties: 
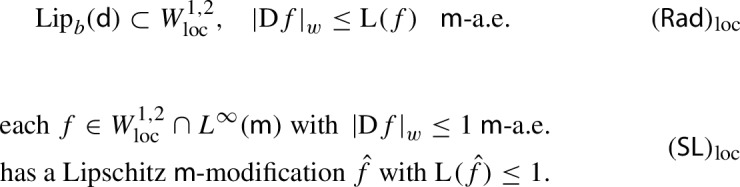


Again by construction, $(\mathsf {Rad})_{\mathrm {loc}}$ holds on every metric measure space.

### Proposition 4.4

Every ${\mathsf {RQCD}}$ space satisfies $(\mathsf {SL})_{\mathrm {loc}}$.

### Proof

Since we already have $(\mathsf {Rad})$, by construction of $\mathsf {Ch}_{{\mathsf {d}},{\mathsf {m}}}$, and , by Corollary 3.7, in order to localize both properties it suffices to show the existence of good Sobolev cut-off functions, similarly to the proof of Theorem 3.9 in [3].

For every $n\in {{\mathbb {N}}}$ and fixed $x_0\in X$ set $\theta _n:x \mapsto n\wedge \big ({2n-{\mathsf {d}}(x,x_0)}\big )_+$. Since ${\mathrm {supp}}\,\theta _n$ is bounded for every $n\in {{\mathbb {N}}}$, we conclude by $(\mathsf {Rad})$ that $\theta _n\in W^{1,2}\cap {\mathcal {C}}_0(X)$ and $\left|\mathrm {D}\theta _n\right|_{w}\le 1$ ${\mathsf {m}}$-a.e. for every $n\in {{\mathbb {N}}}$.

Fix now $f\in W^{1,2}_{\mathrm {loc}}\cap L^\infty ({\mathsf {m}})$ with $\left|\mathrm {D}f\right|_{w}\le 1$ ${\mathsf {m}}$-a.e. Without loss of generality, up to an additive constant, we may assume that $f\ge 0$. By the local property of $\left|\mathrm {D}{\,\cdot \,}\right|_{w}$, we have that

$$\begin{aligned} \left|\mathrm {D}(\theta _n\wedge f)\right|_{w} =&\mathbb {1}_{\left\{ \theta _n\le f\right\} } \left|\mathrm {D}\theta _n\right|_{w}+\mathbb {1}_{\left\{ \theta _n>f\right\} } \left|\mathrm {D}f\right|_{w} \le 1 \quad {\mathsf {m}}\text {-a.e.} \end{aligned}$$

by assumption on *f* and properties of $\theta _n$, whence $\theta _n\wedge f_n\in W^{1,2}$. From  we conclude that $\theta _n\wedge f$ has a non-relabeled ${\mathsf {d}}$-Lipschitz ${\mathsf {m}}$-representative. Analogously, $\theta _{n+1}\wedge f\in W^{1,2}$ and, for all $n\ge \left\Vert f\right\Vert _{L^\infty }$, we have that $\theta _{n+1}\wedge f\equiv \theta _n\wedge f\equiv f$ ${\mathsf {m}}$-a.e. on $B_n(x_0)$. Since ${\mathsf {m}}$ has full topological support, we conclude that the respective ${\mathsf {d}}$-Lipschitz ${\mathsf {m}}$-representatives coincide everywhere on $B_n(x_0)$. As a consequence, $\left( \theta _n\wedge f\right) _n$ is a consistent family of ${\mathsf {d}}$-Lipschitz functions coinciding with *f* ${\mathsf {m}}$-a.e. on $B_n(x_0)$ for all (sufficiently large) $n\in {{\mathbb {N}}}$. The conclusion readily follows letting $n\rightarrow \infty$. $\square$

Denote by $P_t:L^2({\mathsf {m}})\rightarrow L^2({\mathsf {m}})$ the strongly continuous contraction semigroup associated to $(\mathsf {Ch}_{{\mathsf {d}},{\mathsf {m}}}, W^{1,2})$, and, for open sets $A,B\subset X$, set

$$\begin{aligned} P_t(A,B):=\int \limits _A P_t \mathbb {1}_B \mathop {}\!\mathrm {d}{\mathsf {m}}= \int \limits _B P_t \mathbb {1}_A \mathop {}\!\mathrm {d}{\mathsf {m}}. \end{aligned}$$

For sets $A_1,A_2\subset X$ define

$$\begin{aligned} {\mathsf {d}}(A_1,A_2):=\inf _{x_i\in A_i} {\mathsf {d}}(x_1,x_2). \end{aligned}$$

We now give the first application of .

### Theorem 4.5

(Integral Varadhan short-time asymptotics). Every ${\mathsf {RQCD}}$ space satisfies the integral Varadhan-type short-time asymptotic

$$\begin{aligned} \lim _{t\downarrow 0} \big ({-2t \log P_t(U_1,U_2)}\big )= {\mathsf {d}}(U_1,U_2)^2, \quad U_1,U_2 \quad \text {open}. \end{aligned}$$

### Proof

Let $G_\bullet :=\left( G_n\right) _n$ be an increasing exhaustion of *X* consisting of relatively compact open sets. Choosing such $G_\bullet$ in [9, Prop. 2.26] shows that the broad local space ${\mathbb {L}}^{{\mathsf {m}}}_{{\mathrm {loc}},b}$ of bounded functions with bounded $\mathsf {Ch}_{{\mathsf {d}},{\mathsf {m}}}$-energy defined in [9, Sect. 2.6.1] coincides with the local space of bounded functions with bounded $\mathsf {Ch}_{{\mathsf {d}},{\mathsf {m}}}$-energy defined above, viz.

4.2 $$\begin{aligned} {\mathbb {L}}^{{\mathsf {m}}}_{{\mathrm {loc}},b}=\left\{ f\in W^{1,2}_{\mathrm {loc}}\cap L^\infty ({\mathsf {m}}) : \left|\mathrm {D}f\right|_{w}^2\le 1\right\} . \end{aligned}$$

Equation (4.2) shows that—for the form $(\mathsf {Ch}_{{\mathsf {d}},{\mathsf {m}}}, W^{1,2})$—the local Rademacher-type and Sobolev-to-Lipschitz properties defined above respectively coincide with [9, $(\mathsf {Rad}_{{\mathsf {d}},\mu })$ with $\mu :={\mathsf {m}}$ in Dfn. 3.1] and [9, $(\mathsf {SL}_{\mu ,{\mathsf {d}}})$ with $\mu :={\mathsf {m}}$ in Dfn. 4.1]. As a consequence, all the results established in [9] apply as well to present setting.

For a Borel $A\subset X$, let ${{{\bar{{\mathsf {d}}}}}}_{{\mathsf {m}}, A}$ denote the maximal function [9, Prop. 4.14]. By [9, Lem. 4.16] we have

$$\begin{aligned} {\mathsf {d}}({\,\cdot \,}, A)\le {{{\bar{{\mathsf {d}}}}}}_{{\mathsf {m}}, A} \quad {\mathsf {m}}\text {-a.e.}, \quad A \quad \text {Borel}. \end{aligned}$$

By [9, Lem. 4.19, Rmk. 4.19(b)]

$$\begin{aligned} {\mathsf {d}}({\,\cdot \,}, U)\ge {{{\bar{{\mathsf {d}}}}}}_{{\mathsf {m}}, U} \quad {\mathsf {m}}\text {-a.e.} \mathrm {,}\qquad U \quad \text {open}. \end{aligned}$$

Combining the above inequalities thus yields

$$\begin{aligned} {\mathsf {d}}({\,\cdot \,}, U)= {{{\bar{{\mathsf {d}}}}}}_{{\mathsf {m}}, U} \quad {\mathsf {m}}\text {-a.e.}, \quad U \quad \text {open}, \end{aligned}$$

and the conclusion follows as in the proof of [9, Cor. 4.26]. $\square$

Denote again by $P_t:L^\infty ({\mathsf {m}})\rightarrow L^\infty ({\mathsf {m}})$ the extension of $P_t:L^2({\mathsf {m}})\rightarrow L^2({\mathsf {m}})$ to $L^\infty ({\mathsf {m}})$. An ${\mathsf {m}}$-measurable set $A\subset X$ is called *invariant* if $\mathbb {1}_A P_t f\equiv P_t (\mathbb {1}_A f)$ for every $f\in L^\infty ({\mathsf {m}})$. We say that the space $(X,{\mathsf {d}},{\mathsf {m}})$ is *irreducible* if every invariant set is either ${\mathsf {m}}$-negligible or ${\mathsf {m}}$-conegligible.

As a corollary of Theorem 4.5, we obtain the irreducibility of $(X,{\mathsf {d}},{\mathsf {m}})$.

### Corollary 4.6

(Irreducibility). Every ${\mathsf {RQCD}}$ space is irreducible.

### Proof

By [9, Thm. 4.21] for every Borel $A\subset X$ with ${\mathsf {m}}A>0$ there exists a Borel ${\mathsf {m}}$-version ${{\widetilde{A}}}$ of *A* so that the maximal function ${{{\bar{{\mathsf {d}}}}}}_{{\mathsf {m}},A}$ defined in [9, Prop. 4.14] satisfies ${{{\bar{{\mathsf {d}}}}}}_{{\mathsf {m}}, A}= {\mathsf {d}}({\,\cdot \,}, {{\widetilde{A}}})$ ${\mathsf {m}}$-a.e. As a consequence, for every pair of Borel sets $A_1,A_2$ with ${\mathsf {m}}A_1,{\mathsf {m}}A_2>0$, we have that

$$\begin{aligned} {{{\bar{{\mathsf {d}}}}}}_{{\mathsf {m}}}(A_1,A_2):={\mathsf {m}}\text {-}\mathop {{\mathrm{essinf}}}\limits _{y\in A_2}{{{\bar{{\mathsf {d}}}}}}_{{\mathsf {m}}, A_1}(y)\le {\mathsf {d}}({{\widetilde{A}}}_1,{{\widetilde{A}}}_2)<\infty \mathrm {.}\end{aligned}$$

By [4, Prop. 5.1],

$$\begin{aligned} {{{\bar{{\mathsf {d}}}}}}_{{\mathsf {m}}}(A_1,A_2)<\infty \iff P_t(A_1,A_2)>0 \text {\; for every\; } t>0\mathrm {,}\end{aligned}$$

whence

4.3 $$\begin{aligned} P_t(A_1, A_2):=\int \limits _{A_1}P_t\mathbb {1}_{A_2} \mathop {}\!\mathrm {d}{\mathsf {m}}>0 \end{aligned}$$

for every pair of Borel sets $A_1,A_2$ with ${\mathsf {m}}A_1, {\mathsf {m}}A_2>0$. Now, argue by contradiction that $(X,{\mathsf {d}},{\mathsf {m}})$ is not irreducible. Then, there exists a Borel set *A* with ${\mathsf {m}}A, {\mathsf {m}}A^\mathrm {c}>0$ and so that $P_t\mathbb {1}_A=\mathbb {1}_A P_t$. Since (4.3) does not hold for the pair $A^\mathrm {c},A$, we obtain a contradiction, and $(X,{\mathsf {d}},{\mathsf {m}})$ is therefore irreducible. $\square$

### Remark 4.7

(*About irreducibility on* ${\mathsf {RCD}}(K,\infty )$ *spaces*) We stress that, in fact, the same proof of irreducibility presented above for ${\mathsf {RQCD}}$ spaces holds as well on all ${\mathsf {RCD}}(K,\infty )$ spaces, which seems not explicitly stated in the existing literature. The result holds even in the case when ${\mathsf {m}}X=\infty$, since we only rely on the local Rademacher-type and Sobolev-to-Lipschitz properties $(\mathsf {Rad})_{\mathrm {loc}}$ and $(\mathsf {SL})_{\mathrm {loc}}$, which can be proved by the same localization argument as in Proposition 4.4. Note that these arguments hold even if the space $(X,{\mathsf {d}},{\mathsf {m}})$ is not locally compact, in which case the local domain $W^{1,2}_{\mathrm {loc}}$ may be replaced with the broad local domain, see, e.g., [9, Sect. 2.4]. Importantly, this proof does not rely on any heat-kernel estimate.

As the second application, we prove that ${\mathsf {d}}$ coincides with the intrinsic distance—in the sense of e.g. [27, Eq. (1.3)]—associated with the Cheeger energy, viz.

$$\begin{aligned} {\mathsf {d}}_{\mathsf {Ch}_{{\mathsf {d}},{\mathsf {m}}}}(x,y):=\sup \left\{ f(x)-f(y) : f\in W^{1,2}_{\mathrm {loc}}\cap {\mathcal {C}}(X), \left|\mathrm {D}f\right|_{w}^2\le 1 \quad {\mathsf {m}}\text {-a.e.}\right\} . \end{aligned}$$

### Theorem 4.8

Let $(X,{\mathsf {d}},{\mathsf {m}})$ be an ${\mathsf {RQCD}}$ space. Then, $(\mathsf {Ch}_{{\mathsf {d}},{\mathsf {m}}}, W^{1,2})$ is a regular strongly local Dirichlet form on $L^2({\mathsf {m}})$, and

4.4 $$\begin{aligned} {\mathsf {d}}(x,y)&= {\mathsf {d}}_{\mathsf {Ch}_{{\mathsf {d}},{\mathsf {m}}}}(x,y) \end{aligned}$$

4.5 $$\begin{aligned}&= \sup \left\{ f(x)-f(y) : f\in W^{1,2}\cap {\mathcal {C}}_c(X), \left|\mathrm {D}f\right|_{w}^2\le 1 \quad {\mathsf {m}}\text {-a.e.}\right\} . \end{aligned}$$

### Proof

On the one hand, by $(\mathsf {Rad})_{\mathrm {loc}}$ and by a straightforward adaptation of [9, Lemma 3.6], we have that ${\mathsf {d}}\le {\mathsf {d}}_{\mathsf {Ch}_{{\mathsf {m}},{\mathsf {d}}}}$. On the other hand, by $(\mathsf {SL})_{\mathrm {loc}}$ (Proposition 4.4) and by a straightforward adaptation of [9, Prop. 4.2], we have that ${\mathsf {d}}_{\mathsf {Ch}_{{\mathsf {m}},{\mathsf {d}}}}\le {\mathsf {d}}$. Combining the two inequalities shows the equality in (4.4).

Since $\mathsf {Ch}_{{\mathsf {d}},{\mathsf {m}}}$ is regular, and irreducible by Corollary 4.6, $(X,{\mathsf {d}}_{\mathsf {Ch}_{{\mathsf {m}},{\mathsf {d}}}})=(X,{\mathsf {d}})$ satisfies Assumption (A) in [27], and the equality in (4.5) follows by [27, Prop. 1.c, p. 193]. $\square$

### Remark 4.9

Combining Theorem 4.8 with [9, Prop. 2.31] shows that several definitions for the intrinsic distance ${\mathsf {d}}_{\mathsf {Ch}_{{\mathsf {m}},{\mathsf {d}}}}$—and in particular the one of ${\mathsf {d}}_{\mathsf {m}}$ in [9, Dfn. 2.28]—coincide.

In the next corollary, we denote by $p_t$ the density w.r.t. ${\mathsf {m}}$ of the heat-kernel measure of the heat semigroup $P_t$.

### Corollary 4.10

(Pointwise Varadhan short-time asymptotics). Every compact ${\mathsf {RQCD}}_{\mathrm {reg}}$ space satisfies the pointwise Varadhan short-time asymptotics

4.6 $$\begin{aligned} \lim _{t\downarrow 0} \big ({-2t \log p_t(x,y)}\big )= {\mathsf {d}}(x,y)^2 . \end{aligned}$$

### Proof

As a consequence of the ${\mathsf {MCP}}$ condition implicit in the notation for ${\mathsf {RQCD}}_{\mathrm {reg}}$, we have the validity of the local weak 2-Poincaré inequality, see [29, Cor. 6.6(ii)], and of the local doubling property, as noted in Corollary 2.8. Both properties are in fact global, by compactness of *X*. By Proposition 4.2 and Theorem 4.8, the Dirichlet form $(\mathsf {Ch}_{{\mathsf {d}},{\mathsf {m}}},W^{1,2})$ is *strongly regular*, i.e. regular and so that the intrinsic distance ${\mathsf {d}}_{\mathsf {Ch}_{{\mathsf {d}},{\mathsf {m}}}}$ induces the original topology. By [24, Thm. 4.1] we conclude (4.6) with ${\mathsf {d}}_{\mathsf {Ch}_{{\mathsf {d}},{\mathsf {m}}}}$ in place of ${\mathsf {d}}$. The conclusion now follows since ${\mathsf {d}}={\mathsf {d}}_{\mathsf {Ch}_{{\mathsf {d}},{\mathsf {m}}}}$ by Theorem 4.8. $\square$

Finally let us briefly discuss the Lipschitz regularization property of the semigroup $\left( P_t\right) _{t\ge 0}$. Let $\mathsf{c}: [0,+\infty ) \rightarrow (0, +\infty )$ be a measurable function so that locally uniformly bounded away from 0 and infinity. Following [26, Definition 3.4], we say that an ${\mathsf {RQCD}}$ space satisfies ${\mathsf {BE}}_w({\mathsf {c}}, \infty )$ if for all $f \in W^{1,2}$ and $t \ge 0$, 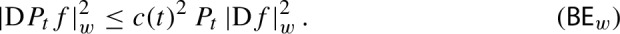


### Corollary 4.11

($L^\infty$-to-${{\mathrm{Lip}}}_{{\textit{b}}}$-Feller). Assume that $(X,{\mathsf {d}},{\mathsf {m}})$ is an ${\mathsf {RQCD}}$ space satisfying (${\mathsf {BE}}_w$). Then, $P_t: L^\infty (X, {\mathsf {m}}) \rightarrow {{\mathrm{Lip}}}_{{\textit{b}}}(X, {\mathsf {d}})$ for any $t>0$ and

$$\begin{aligned} \sqrt{2I_{-2}(t)}\, \mathrm {L}_{{\mathsf {d}}}(P_tf) \le \left\Vert f\right\Vert _{L^\infty }, \quad I_{-2}(t):=\int \limits _0^t{\mathsf {c}}^{-2}(s)\mathop {}\!\mathrm {d}s. \end{aligned}$$

### Proof

By Theorem 3.6, the space satisfies , i.e. [26, (P.5)], and the assertion holds by [26, Cor. 3.21]. $\square$
